# Supplementary material for: Allele-specific splicing modulates protein isoforms and Alzheimer’s risk
Source: bioRxiv. 2026 Feb 16:2026.02.13.705796. Preprint. [Version 1] doi: 10.64898/2026.02.13.705796 (PMC12934767; doi:10.64898/2026.02.13.705796)
Supplement: Supplement 1 [file media-1.pdf]

Figure S1

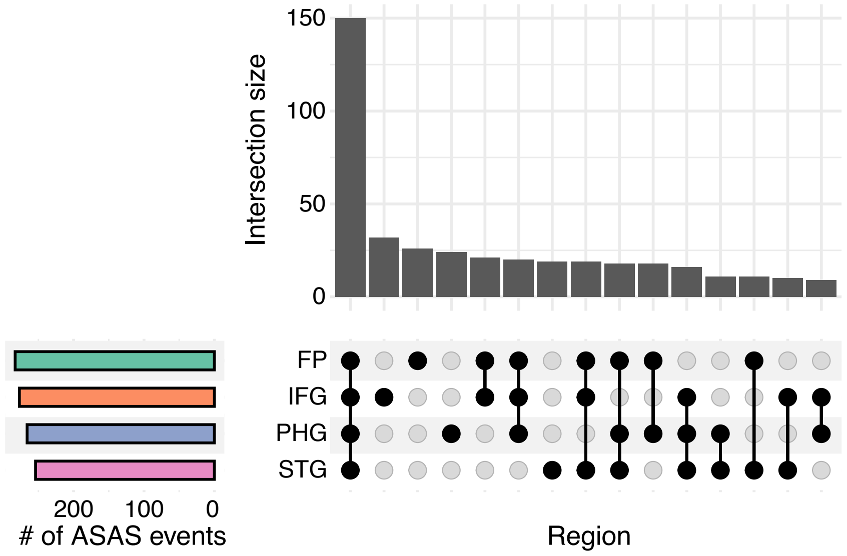

Figure S1. Overlap of testable ASAS events across brain regions.
Overlap of ASAS events that were testable in at least one sample per brain region across brain regions. STG: superior temporal gyrus; PHG: parahippocampal gyrus; IFG: inferior frontal gyrus; FP: frontal pole.

Figure S2

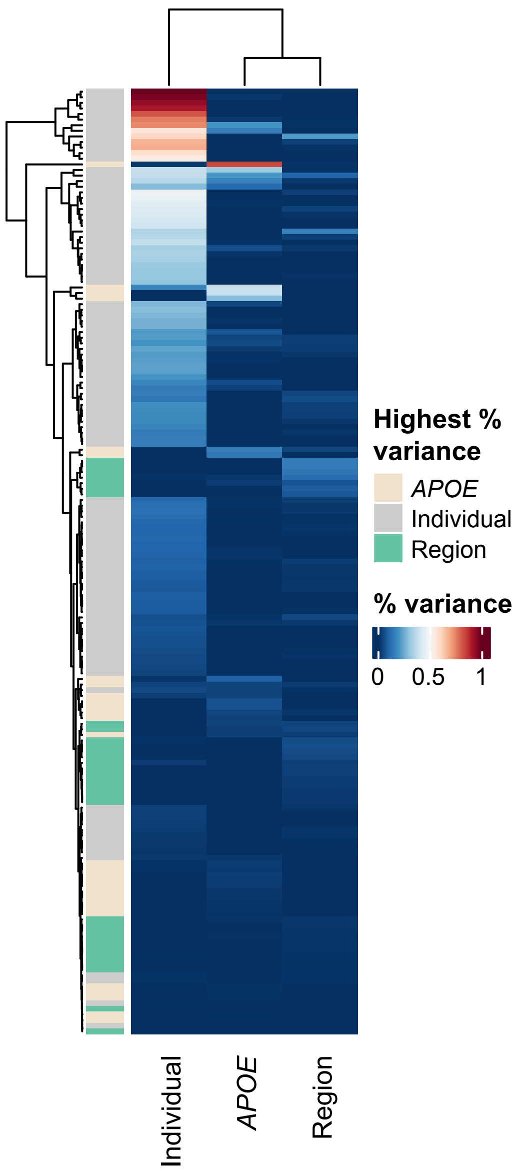

% variance from APOE	Gene	Chromosome	AS_region
84.04%	*VSTM2A-OT1*	chr7	54557850:54559971
38.65%	*FAM86FP*	chr12	8234276:8234426
38.47%	*ANKRD36*	chr2	97163244:97163854
28.36%	***RNF19A***	chr8	100300528:100300651
15.73%	*SNHG14*	chr15	25204684:25204728
13.90%	*ACADM*	chr1	75733051:75733149
9.26%	*ZNF638*	chr2	71393394:71393645
6.31%	*PPP1R9A*	chr7	95274085:95274168
6.08%	*SNHG14*	chr15	25065122:25065454
4.11%	***ACSL6***	chr5	131991745:131991813
3.89%	*GET1-SH3BGR*	chr21	39499823:39499915
3.82%	*SDR39U1*	chr14	24441674:24441795
3.36%	*ZNF91*	chr19	23374638:23374764
3.00%	*NEBL*	chr10	20850395:20850502
2.52%	***RIMS2***	chr8	104094478:104094729
2.34%	***TULP4***	chr6	158501678:158504178
2.28%	*STX16-NPEPL1*	chr20	58691405:58691562
2.08%	*ZBTB47-AS1*	chr3	42612292:42612367
1.92%	*ENSG00000268575*	chr1	1704223:1704344
1.60%	*LRRC8C-DT*	chr1	89587903:89588085
1.52%	*NAP1L4*	chr11	2989129:2989271
1.14%	***KIF1B***	chr1	10303163:10306464
1.06%	*HECTD1*	chr14	31128612:31129448
0.79%	***WASF3***	chr13	26676549:26676724
0.37%	***PHF14***	chr7	10982372:10983159
0.35%	*ADD1*	chr4	2867972:2868129
0.30%	*PDE4DIP*	chr1	149012591:149012776
0.16%	*RNF157-AS1*	chr17	76153125:76153788

Figure S2. Contribution of variance from brain regions, individuals, and *APOE* genotype to ASAS patterns.
The percent of variance a brain region (Region), individual, or *APOE* genotype (*APOE*) contributes to for ASAS events for exons identified in at least 2 individuals per brain region and 2 brain regions per individual (Methods). Each row represents an exon, and the columns represent the percent variance of the different categories. Exons are colored by which category the highest percent variance was from. Exons with the highest percent variance from the *APOE* genotype and the percent variance greater than 0.1% are shown in the table. AD-relevant gene names are in bold. Ensembl IDs are shown for genes without approved HGNC symbols. All coordinates are from the hg38 human genome assembly.

Figure S3

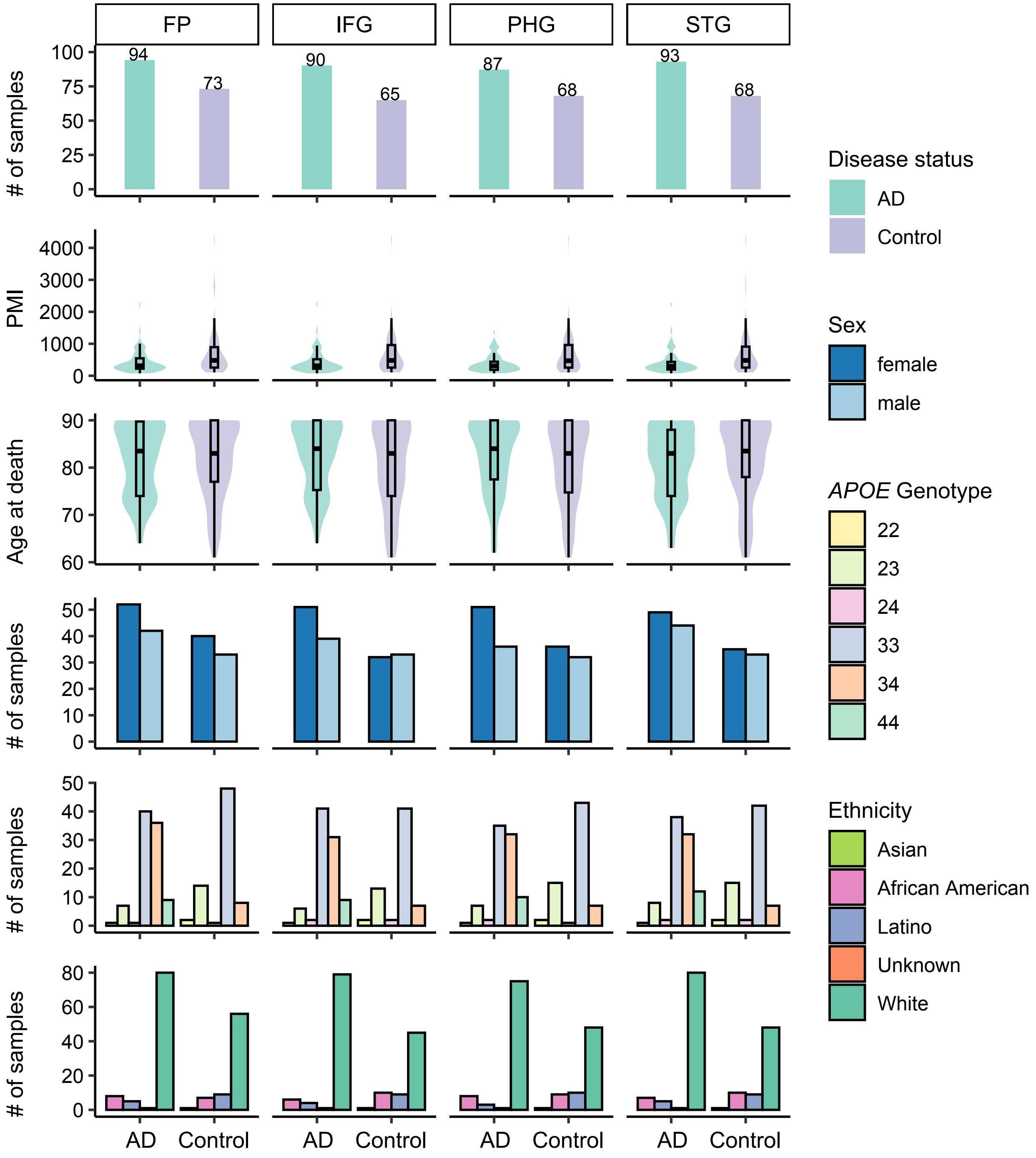

Figure S3. Distribution of sample size and cohort demographics for identifying putative functional SNPs.
Sample size, post mortem interval (PMI), age at death, sex, *APOE* genotype, and ethnicity distributions for each brain region in AD and Control groups, based on "definite AD" and "normal" CERAD scores, respectively (Methods). STG: superior temporal gyrus; PHG: parahippocampal gyrus; IFG: inferior frontal gyrus; FP: frontal pole.

Figure S4

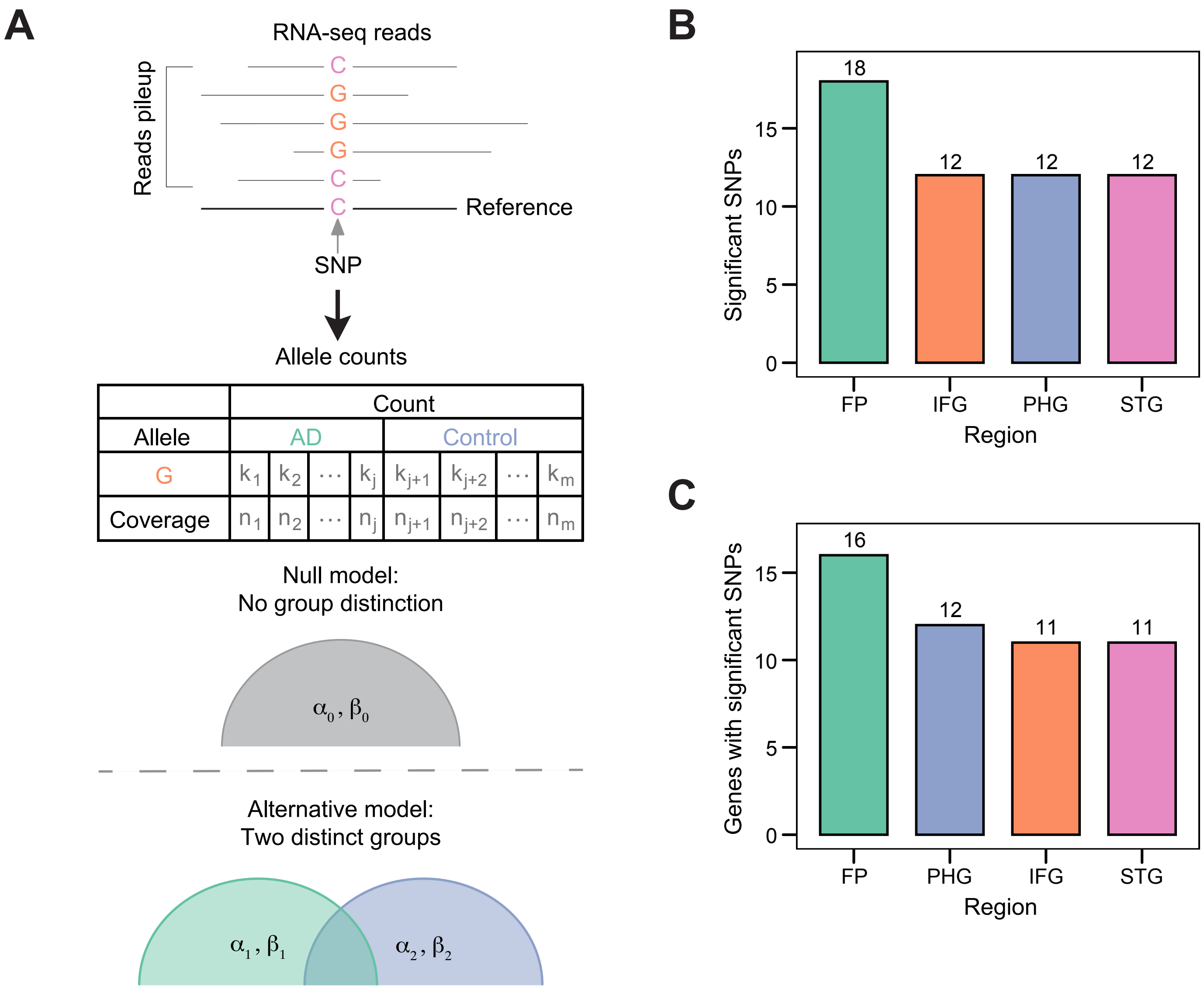

Figure S4. Identification of tag SNPs displaying differential allelic bias in AD.
(A) Schematic for identifying differential allelic bias of tag SNPs (Methods). The allelic ratio was modeled as a beta-binomial distribution using a likelihood ratio test to determine significance between AD and control groups. (B) Number of significant differential tag SNPs identified in each region. (C) Number of genes with at least one significant differential tag SNP identified in each region. FP: Frontal Pole; PHG: Parahippocampal Gyrus; STG: Superior Temporal Gyrus; IFG: Inferior Frontal Gyrus.

Figure S5

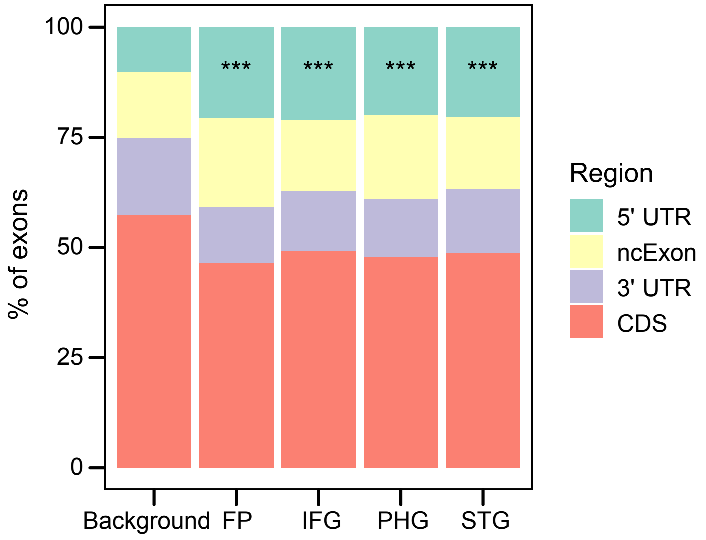

Figure S5. 5’ UTR enrichment for ASAS exons in each brain region.
Genomic regions overlapping ASAS exons in each brain region compared to all annotated exons (Background). ***p < 0.001 (Fisher’s exact test). CDS: coding sequence. ncExon: exons in non-coding transcripts. FP: Frontal Pole; PHG: Parahippocampal Gyrus; STG: Superior Temporal Gyrus; IFG: Inferior Frontal Gyrus.

Figure S6

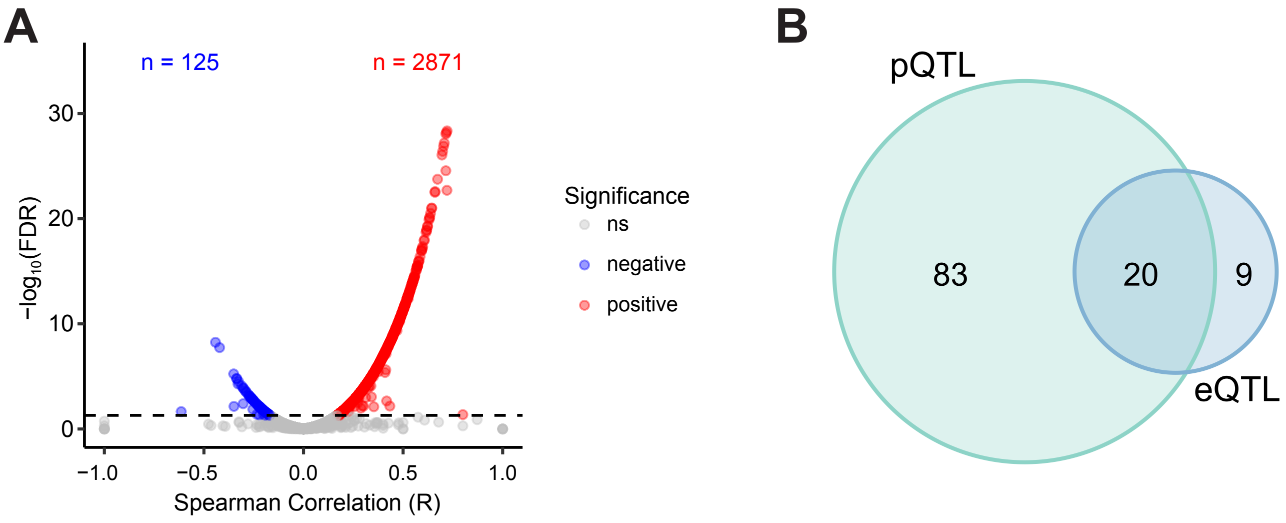

Figure S6. Relationship between mRNA and protein abundance
(A) Significance and directionality (Spearman correlation coefficient) of the correlation between protein abundance and RPKM for each gene (Methods). Each dot represents one gene. Red and blue colors represent genes with significant mRNA and protein abundance correlation, in the positive and negative direction, respectively (FDR ≤ 0.05). ns: not significant. (B) Overlap of 5' UTR ASAS-associated SNPs and pQTLs (Methods) or GTEx brain eQTLs within the same gene.
